# Effects of a previously selected antibiotic resistance on mutations acquired during development of a second resistance in *Escherichia coli*

**DOI:** 10.1186/s12864-019-5648-7

**Published:** 2019-04-11

**Authors:** Marloes Hoeksema, Martijs J. Jonker, Stanley Brul, Benno H. ter Kuile

**Affiliations:** 1 Laboratory for Molecular Biology and Microbial Food Safety, Swammerdam Institute for Life Sciences, University of Amsterdam, Amsterdam, The Netherlands; 2 RNA Biology & Applied Bioinformatics, Swammerdam Institute for Life Sciences, University of Amsterdam, Amsterdam, The Netherlands; 3 Netherlands Food and Consumer Product Safety Authority, Office for Risk Assessment, Utrecht, The Netherlands

**Keywords:** Bactericidal antibiotics, de novo resistance, Mutations, Whole genome sequencing

## Abstract

**Background:**

The effect of mutations conferring antibiotic resistance can depend on the genetic background. To determine if a previously de novo acquired antibiotic resistance influences the adaptation to a second antibiotic, antibiotic resistance was selected for by exposure to stepwise increasing sublethal levels of amoxicillin, enrofloxacin, kanamycin, or tetracycline. *E. coli* populations adapted to either a single or two antibiotics sequentially were characterized using whole genome population sequencing and MIC measurements.

**Results:**

In a wild-type background, adaptation to any of the antibiotics resulted in the appearance of well-known mutations, as well as a number of mutated genes not known to be associated with antibiotic resistance. Development of a second resistance in a strain with an earlier acquired resistance to a different antibiotic did not always result in the appearance of all mutations associated with resistance in a wild-type background. In general, a more varied set of mutations was acquired during secondary adaptation. The ability of *E. coli* to maintain the first resistance during this process depended on the combination of antibiotics used. The maintenance of mutations associated with resistance to the first antibiotic did not always predict the residual MIC for that compound.

**Conclusions:**

In general, the data presented here indicate that adaptation to each antibiotic is unique and independent. The mutational trajectories available in already resistant cells appear more varied than in wild-type cells, indicating that the genetic background of *E. coli* influences resistance development. The observed mutations cannot always fully explain the resistance pattern observed, indicating a crucial role for adaptation on the gene expression level in de novo acquisition of antibiotic resistance.

## Background

To be able to predict, prevent, or slow down development of antibiotic resistance, the molecular mechanisms that drive development of antibiotic resistance need to be understood. Antibiotic resistance can develop in three distinct ways: through horizontal gene transfer, chromosomal mutations, or phenotypic adaptation. In *Escherichia coli*, the first stages of de novo development of antibiotic resistance occur on a phenotypic level, controlled by adaptation on the gene expression level [1]. After this initial stage, mutations appear that, in most cases, confer a reduction in bacterial fitness [1–3]. Often, this is followed by the appearance of compensatory mutations that reduce the loss in fitness without reducing the acquired resistance [4].

Antibiotic resistance can be selected for by exposure to stepwise increasing sublethal levels of the antibiotic [5]. Although each antibiotic class has a specific cellular target, the radical-based theory suggests that as a secondary effect increased levels of cellular reactive oxygen species (ROS) occur as a result of a pathway common to all bactericidal antibiotics [6, 7]. Following this logic, similar mutations might occur in cells with acquired resistance to different bactericidal antibiotics. During evolution experiments exposing *E. coli* to steadily increasing levels of antibiotics, cells with an earlier acquired resistance to a bactericidal antibiotic adapt to a second bactericidal, but not bacteriostatic, antibiotic at a more rapid rate, corroborating this theory [8].

The effect of resistance associated mutations varies depending on the genetic background, such as the presence of other resistance mutations, a phenomenon known as epistasis [9, 10]. The fitness cost incurred by mutations determines the evolutionary pathways available [11], suggesting that the evolutionary history of bacteria may influence the type or number of mutations that are acquired upon exposure to an antibiotic. In this study, we investigate if a previously de novo acquired antibiotic resistance influences the adaptation to a second antibiotic. Whole genome population sequencing was applied to *E. coli* strains with de novo acquired resistance to either one or to two antibiotics sequentially. We previously reported on larger genomic changes that occur during adaptation to a single antibiotic, or two antibiotics successively [12]. In this study, we provide a comprehensive overview of the different mutations that are acquired when wild-type or antibiotic-resistant *E. coli* is exposed to amoxicillin, enrofloxacin, kanamycin, or tetracycline.

## Results

**Fig. 1:**
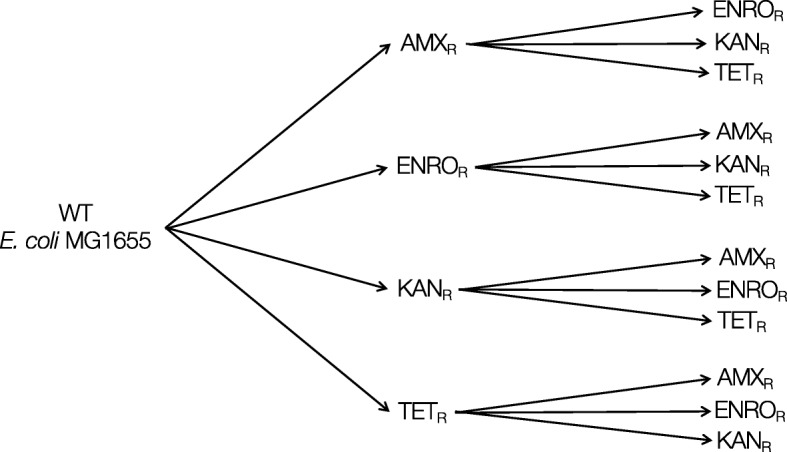
Set-up of evolution experiments inducing resistance. Duplicate strains of wild-type *E. coli* MG1655 were made resistant by exposure to increasing non-lethal concentrations of amoxicillin (AMX), enrofloxacin (ENRO), kanamycin (KAN), or tetracycline (TET). Two replicates of each of the primary resistant strains were subsequently made resistant to one of the three other antibiotics using the same approach. As a result 4 replicates were obtained of all double exposed strains

This study addressed three questions: 1) which mutations are associated with the development of resistance by *E. coli* wild-type against specific antibiotics? 2) Are the same mutations observed when cells made resistant against one antibiotic become resistant to a second one? 3) Are initial mutations lost when cells resistant to one antibiotic are made resistant to another one? To answer these questions, strains exposed to only amoxicillin, enrofloxacin, or kanamycin, all bactericidal antibiotics from different classes, or the bacteriostatic antibiotic tetracycline were compared to cells made resistant to two of these antibiotics successively. Resistant strains were generated by exposing wild-type *E. coli* or a strain with an earlier acquired resistance to increasing but sublethal concentrations of any of these antibiotics [8]. The initial resistance was selected for in duplicate strains. Two replicates of each of these strains were made resistant to a second antibiotic, resulting in four strains with the same exposure history (Fig. 1). Genomic DNA was isolated from the entire population at selected time points for whole genome sequencing at an average read depth of 226 to identify genetic changes associated with acquired resistance to a single and to subsequent antibiotics.

Adaptation of the wild-type to amoxicillin (Table 1) resulted in two well-scrutinized mutations in the *ampC* promoter [1, 13], and in mutations in *ompR*, *citF*, and *rpoD,* all with a population frequency of 1. One of the replicate strains acquired a mutation in *waaB* in addition to these mutations. In the wild-type strain adapted to enrofloxacin, either ten or eleven genes were mutated, and a low-frequency mutation occurred in *mutL*. Mutations in *gyrA*, *parC*, *parE,* soxR, and *acrR*, commonly associated with resistance to fluoroquinolones [14–16] were accompanied by less frequently observed mutations in the genes *proY* and *yciO*. In addition, mutations in the 5′ UTR of *rpsJ*, *ybjL* or *ybjM*, and *gtlB* were detected. When wild-type cells were exposed to kanamycin, mutations accumulated in *sbmA*, *sapF*, *cpxA*, *npr*, *fusA*, and the 5′ UTR of *uidA*. Interestingly, a mutation in *gyrB*, most often associated with resistance to fluoroquinolones [17], was identified with a population frequency of 1 in both wild-type-adapted strains. Development of resistance to tetracycline in wild-type *E. coli* resulted in mutations in *acrR* and *acrB*, *ompF*, *mlaA*. A mutation in *rpoC* was only detected in a strain with partial resistance to tetracycline.

**Table 1:** Mutations associated with resistance to amoxicillin, enrofloxacin, kanamycin, or tetracycline in wild-type *E. coli*

Exposure to amoxicillin					
			WT		WT
Gene product	Gene	Mutation	40 μg/ml	1280 μg/ml	1280 μg/ml
B-lactamase	*ampC*	G23 T (promoter)	1	1	1
		C-11 T (promoter)	1	1	1
Transcriptional regulator	*ompR*	E88A		1	1
Citrate lyase	*citF*	G432A		1	0.97
Sigma 70	*rpoD*	D445V		0.96	1
UDP-D-galactose	*waaB*	Del C3803246 (FS297)			1
Exposure to enrofloxacin					
				WT	WT
Gene product	Gene	Mutation		1024 μg/ml	1024 μg/ml
DNA gyrase subunit A	*gyrA*	D87G		1	
		S83 L		1	1
		D87N			1
DNA gyrase subunit B	*gyrB*	S463F			1
DNA topoisomerase IV subunit A	*parC*	S80R		1	1
DNA topoisomerase IV subunit B	*parE*	Ins Q458		0.94	1
Cryptic proline/histidine transporter	*proY*	A235S		1	1
Transcriptional regulator	*acrR*	Del 485,885–11 (FS42)		1	1
Transcriptional regulator	*soxR*	T133S, del 4,277,899–903 (FS144)		1	1
S10 subunit of 30S	*rpsJ*	5′ UTR (G3453306 T)		1	1
Putative transport protein/Putative inner membrane protein	ybjL/ybjM*	5′ UTR (A889923G)		1	1
Glutamate synthase subunit	*gltB*	5′ UTR (C3354487T)		1	1
Putative RNA binding protein	*yciO*	N64Y		1	1
Mismatch repair protein	*mutL*	W390 L			0.12
Exposure to kanamycin					
			WT		WT
Gene product	Gene	Mutation	32 μg/mL	1024 μg/mL	1024 μg/mL
Peptide antibiotic transporter	*sbmA*	Del 397,306–27 (FS 222)		1	1
ATP binding protein of putrescine ABC exporter	*sapF*	L181Q		1	1
Sensor protein of Cpx TCS	*cpxA*	Q242L		1	1
DNA gyrase subunit B	*gyrB*	S464Y		1	1
Phosphorelay protein	*npr*	L23R		1	1
Elongation factor G	*fusA*	Q242L		1	
		A608V	0.19		
		F05 L	0.74		1
β-D-glucuronidase	*uidA*	5′ UTR (T1696220C)			1
Exposure to tetracycline					
			WT		
Gene product	Gene	Mutation	16 μg/mL	64 μg/mL	
Multidrug efflux pump RND permease	*acrB*	I45L	1	1	
Transcriptional regulator	*acrR*	P85Q	1	1	
Outer membrane porin F	*ompF*	T71S	0.65	0.04	
		Ins 7 nt after G986771 (FS71)		0.60	
Outer membrane lipoprotein	*mlaA*	Del N41/F42		0.9	
RNA polymerase subunit β’	*rpoC*	G367C	0.34		

Numbers shown indicate frequency of mutation in population. *Del* = deletion, *In*s = insertion, underlined letters and numbers indicate nucleotides and their genomic position, * indicates that mutation could affect either gene. For amoxicillin, enrofloxacin, and kanamycin, two independent strains were sequenced

**Fig. 2:**
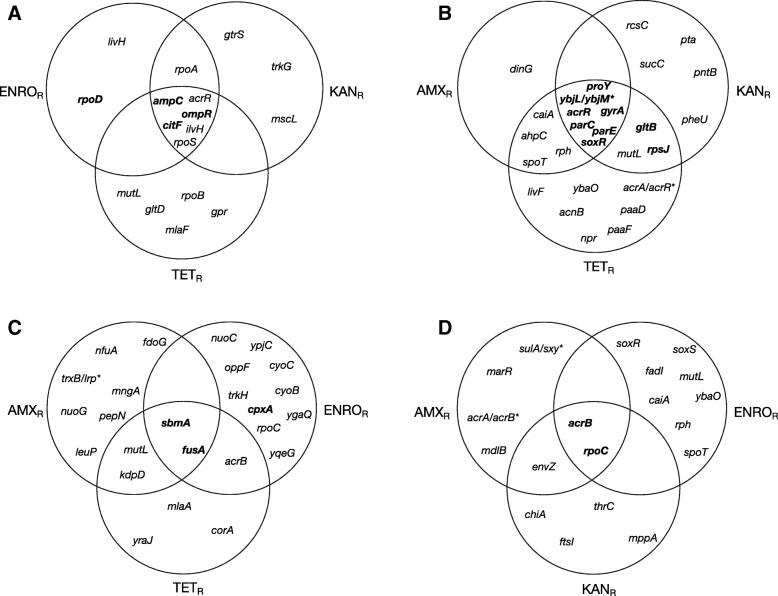
Overlap of mutations associated with resistance to amoxicillin (**a**), enrofloxacin (**b**), kanamycin (**c**), or tetracycline (**d**) in strains with a previously acquired resistance to a different antibiotic. AMX_R_: amoxicillin resistant, ENRO_R_: enrofloxacin resistant, KAN_R_: kanamycin resistant, TET_R_: tetracycline resistant. Mutations associated with resistance in a wild-type background are shown in bold. Mutations associated with the previous resistance have been omitted from the figure

Development of secondary resistance does not always result in the appearance of all mutations associated with resistance to that specific antibiotic in a wild-type strain (Fig. 2, Tables 2, 3, 4 and 5). This is most apparent in the strains adapted to kanamycin (Fig. 2c), where only mutations in *sbmA* and *fusA* are acquired by all strains. A high degree of variability can be observed between the replicates, as indicated by the frequencies displayed in Tables 2, 3, 4 and 5(column headed “Strains”), suggesting that secondary adaptation allows for more flexibility than primary development of resistance. Moreover, selected resistance to a second antibiotic results in the appearance of mutations that are acquired by multiple strains already resistant to another antibiotic but not by wild-type strains, such as *rpoA* during secondary amoxicillin resistance development (Fig. 2a), *caiA*, *ahpC*, *rph*, and *spoT* during adaptation to enrofloxacin (Fig. 2b), *kdpD* and *acrB* during development of resistance to kanamycin as a second antibiotic (Fig. 2c), or *envZ* when already resistant cells adapt to tetracycline (Fig. 2d). Within different antibiotic-resistant populations, a *mutL* mutation was identified in several samples, with a population frequency varying from 0.09 to 0.20.

**Table 2:** Mutations associated with resistance to amoxicillin in strains with a previously acquired resistance to enrofloxacin, kanamycin, or tetracycline

Gene product	Gene	Mutation	Pop. frequency	Strains
Transcriptional regulator	*acrR*	L204R, Del C205	0.98–1	E_1/4_, K_2/4_ T_2/4_
B-lactamase	***ampC***	C35A (promoter)	1	E_4/4,_ K_3/4,_ T_1/4_
		C-11 T (promoter)	1	E_4/4,_ K_3/4,_ T_1/4_
		Ins T after C4379837 (promoter)	1	K_1/4_
		Ins T after A4379015 (promoter)	1	T_1/4_
Citrate lyase	***citF***	G432A	1	E_4/4_, K_3/4_, T_1/4_
Glutamate synthase subunit	*gltD*	A80A	0.18	T_1/4_
L-glyceraldehyde 3-phosphate reductase	*gpr*	L180F	1	T_2/4_
CPS-53 glucosyl transferase	*gtrS*	V351 V	0.96	K_1/4_
Acetolactate synthase/acetohydroxybutanoate synthase subunit	*ilvH*	G52C	0.60–1	E_2/4_, K_2/4_, T_1/4_
Branched chain amino acid/phenylalanine ABC transporter membrane subunit	*livH*	G239R	0.18	E_1/4_
Intermembrane phospholipid transport system subunit	*mlaF*	Ins AIT between T38-A39	0.65–0.66	T_2/4_
Large conductance mechanosensitive channel	*mscL*	L45Q	0.98	K_1/4_
Mismatch repair protein	*mutL*	W390 L	0.11–0.2	T_2/4_
Transcriptional regulator	***ompR***	E88A	1	E_4/4_, K_3/4_, T_1/4_
RNA polymerase subunit α	*rpoA*	C269S	1	E_1/4_, K_1/4_
RNA polymerase subunit β	*rpoB*	E564A	0.15	T_1/4_
Sigma 70	***rpoD***	D445V	1	E_4/4_
Alternative sigma factor σS	*rpoS*	E16Stop	0.96–1	K_2/4_, T_1/4_
		Del Q59-E330	0.91–1	T_2/4_
Potassium ion transporter	*trkG*	G159D	1	K_1/4_

*Del* = deletion, *Ins* = insertion, underlined letters and numbers indicate nucleotides and their genomic position. Population frequency indicates the mutational frequency in the sequenced population. Strains indicates the prevalence of a mutation within different replicates. E: population with previously acquired resistance to enrofloxacin, K: population with previously acquired resistance to kanamycin, T: population with previously acquired resistance to tetracycline. X/Y: X indicates number of replicates with particular mutation, Y indicates total number of replicates that were sequenced. Mutations associated with resistance in a wild-type background are shown in **bold**. Mutations associated with the previous resistance have been omitted from the table

**Table 3:** Mutations associated with resistance to enrofloxacin in strains with a previously acquired resistance to amoxicillin, kanamycin, or tetracycline

Gene product	Gene	Mutation	Pop. frequency	Strains
Multidrug efflux pump membrane fusion lipoprotein/transcriptional regulator	acrA/acrR*	5′ UTR (Del 485,695–711)	1	T_1/3_
Transcriptional regulator	***acrR***	Del 485,885–11 (FS42)	1	A_3/4_, K_1/4_, T_2/3_
Aconitate hydratase B/2-methylisocitrate dehydratase	*acnB*	G362 V, Del L363-V865	1	T_1/3_
Alkyl hydroperoxide reductase	*ahpC*	Del F38	0.40–1	A_2/4_, T_1/3_
Crotonobetainyl-CoA reductase	*caiA*	C154A, F155P, I156T, T157P, S158R, S159 T, A160S, Y161W, Del T162-R380	1	A_2/4_, T_1/4_
ATP-dependent helicase	*dinG*	Del Q641-R716	0.89	A_1/4_
Glutamate synthase subunit	***gltB***	5′ UTR (C3354487T)	1	K_1/4_, T_2/3_
DNA gyrase subunit A	***gyrA***	S83 L	1	A_4/4_, E_1/4_, T_3/3_
		D87G	1	A_3/4_, K_2/4_, T_1/3_
		D87N	1	K_2/4_, T_2/3_
Branched chain amino acid/phenylalanine ABC transporter membrane subunit	*livF*	G239F	0.87	T_1/4_
Mismatch repair protein	*mutL*	W390 L	0.09–0.14	K_1/4_, T_2/3_
Phosphorelay protein	*npr*	L23R	1	T_1/3_
Phenylacetate degradation protein	*paaD*	A8S	1	T_1/3_
Putative 2,3-dehydroadipyl-CoA hydratase	*paaF*	V35 L	1	T_1/3_
DNA topoisomerase IV subunit A	*parC*	S80R	0.94–1	A_3/4_, K_4/4_, T_3/3_
DNA topoisomerase IV subunit B	*parE*	Ins Q458	0.97–1	A_3/4_, K_2/4_, T_2/3_
		E459Q	0.03	A_1/4_
		S425P	0.81	K_2/4_
		Ins S after A457	0.95	T_1/3_
tRNA-Phe	*pheU*	5′ UTR (G4362633 T)	1	K_1/4_
Cryptic proline/histidine transporter	***proY***	A235S	1	A_3/4_, K_1/4_, T_2/3_
Pyridine nucleotide transhydrogenase subunit β	*ptnB*	V434 V	0.57	K_1/4_
Phosphate acetyltransferase	*pta*	Del T431-Q714	0.56	K_1/4_
Sensory histidine kinase	*rcsC*	E44K	1	K_2/4_
Truncated RNase PH	*rph*	Del R676	1	A_2/4_, T_1/3_
S10 subunit of 30S	***rpsJ***	5′ UTR (G3453306 T)	1	K_1/4_, T_2/3_
Bifunctional (p) ppGpp synthase/hydrolase	*spoT*	Ins C after T3815879 (FS223)	1	A_2/4_, T_1/4_
Transcriptional regulator	***soxR***	T113S, del 4,277,899–903 (FS14)	1	A_3/4_, K_1/4_, T_1/3_
		Ins G136	0.15	K_1/4_
		L139F, del T after C4277882	0.85	K_1/4_
		A146E	1	K_1/4_
		Del R139-N154	1	K_1/4_
Succinyl-CoA synthetase subunit β	*sucC*	Del A218-N221	0.51	K_1/4_
DNA-binding transcriptional activator	*ybaO*	T33P	1	T_1/4_
Putative RNA binding protein	***yciO***	N64Y	1	A_1/4_
Putative transport protein/Putative inner membrane protein	***ybjL/ybjM*** *	5′ UTR (A889923G)	1	A_3/4_, K_1/4_, T_2/3_

*Del* = deletion, *Ins* = insertion, underlined letters and numbers indicate nucleotides and their genomic position, * indicates that mutation could affect either gene. Population frequency indicates the mutational frequency in the sequenced population. Strains indicates the prevalence of a mutation within different replicates. E: population with previously acquired resistance to enrofloxacin, K: population with previously acquired resistance to kanamycin, T: population with previously acquired resistance to tetracycline. X/Y: X indicates number of replicates with particular mutation, Y indicates total number of replicates that were sequenced. Mutations associated with resistance in a wild-type background are shown in **bold**. Mutations associated with the previous resistance have been omitted from the table

**Table 4:** Mutations associated with resistance to kanamycin in strains with a previously acquired resistance to amoxicillin, enrofloxacin, or tetracycline

Gene product	Gene	Mutation	Pop. frequency	Strains
Multidrug efflux pump RND permease	*acrB*	V139F	0.73–1	E_2/3_
Inner membrane magnesium ion transporter	*corA*	Del V264-F266	0.93	T_1/4_
Sensor protein of Cpx two-component system	***cpxA***	Q242L	1	E2/3
Cytochrome bo3 ubiquinol oxidase subunit 1	*cyoB*	G662C	0.86–0.93	E_2/3_
		Del G448658 (FS661)	0.13	E_1/3_
		Start1L	0.12	E_1/3_
Cytochrome bo3 ubiquinol oxidase subunit 3	*cyoC*	Start1I	0.86–0.93	E_2/3_
		Del C448658 (FS1)	0.13	E_1/3_
		N661Stop	0.12	E_1/3_
Formate dehydrogenase O subunit α	*fdoG*	V226 L	0.36–0.58	A_2/3_
Elongation factor G	***fusA***	T393I	1	A1/3
		F605 L	1	E3/3, T3/4
		P610L	0.92–0.97	A2/3
		P610T	0.96	T1/4
sensory histidine kinase	*kdpD*	Q728P	0.36–0.61	A_1/3_, T_1/4_
tRNA-Leu	*leuP*	V16 V	0.67	A_1/3_
Outer membrane lipoprotein	*mlaA*	Q728P	0.88	T_1/4_
		Del F42-N43	0.92	T_1/4_
2-O-α-mannosyl-D-glycerate specific PTS permease	*mngA*	F431 L	0.28–0.59	A_2/3_
Mismatch repair protein	*mutL*	W390 L	0.11–0.13	A_1/4_, T_2/4_
Iron-sulfur cluster carrier protein	*nfuA*	Ins 8 nt after G3546085 (FS154)	0.23	A_1/3_
NADH:quinone oxidoreductase subunit CD	*nuoC*	Del R471-L474	0.93	E_1/3_
NADH:quinone oxidoreductase subunit G	*nuoG*	Ins C after A2399396 (FS257)	0.4	A_1/3_
Murein/oligopeptide ABC transporter subunit	*oppF*	L888Stop	0.74–1	E_2/3_
		Ins SIQ after L187	0.14	E_1/3_
Aminopeptidase N	*pepN*	V809E	0.57	A_1/3_
RNA polymerase subunit β’	*rpoC*	G367C	0.16	E_1/3_
ATP binding protein of putrescine ABC exporter	***sapF***	L181Q	1	T2/4
Peptide antibiotic transporter	***sbmA***	Ins G after T393897 (FS88)	0.99	E_1/3_
		W98Stop	0.44	A_1/3_
		Y162Stop	0.11	T_1/4_
		W179Stop	0.53–0.60	T_2/4_
		S250Stop	0.44	A_1/3_
		L369 L	0.14	T_2/4_
		I370L	0.14	T_2/4_
Uncharacterized protein	*ygaQ*	Q417K	1	E_2/3_
Uncharacterized protein	*ypjC*	5′ UTR (T2785462C)	1	E_2/3_
Putative transporter	*yqeG*	P282P	0.22	E_1/3_
Putative fimbrial usher protein	*yraJ*	Q614L	0.18	T_1/4_
		H615H	0.19	T_1/4_

*Del* = deletion, *Ins* = insertion, underlined letters and numbers indicate nucleotides and their genomic position. Population frequency indicates the mutational frequency in the sequenced population. Strains indicates the prevalence of a mutation within different replicates. A: population with previously acquired resistance to amoxicillin, E: population with previously acquired resistance to enrofloxacin, T: population with previously acquired resistance to tetracycline. X/Y: X indicates number of replicates with particular mutation, Y indicates total number of replicates that were sequenced. Mutations associated with resistance in a wild-type background are shown in **bold**. Mutations associated with the previous resistance have been omitted from the table

**Table 5:** Mutations associated with resistance to tetracycline in strains with a previously acquired resistance to amoxicillin, enrofloxacin, or kanamycin

Gene product	Gene	Mutation	Pop. frequency	Strains
Multidrug efflux pump membrane fusion lipoprotein/multidrug efflux pump RND permease	acrA/acrB*	5′ UTR (Del 485,695–711)	0.77	A_1/4_
Multidrug efflux pump RND permease	***acrB***	V139F	1	A_1/4_, E_4/4_
		S665A	1	A_1/4_
Transcriptional regulator	***acrR***	I45L	0.97	K_1/1_
		P85Q	1	A_3/4_, K_1/4_
		E130K	1	K_1/1_
		R442Q	1	K_1/1_
Crotonobetainyl-CoA reductase	*caiA*	C154A, F155P, I156T, T157P, S158R, S159 T, A160S, Y161W, Del T162-R380	1	E_1/4_
Endochitinase	*chiA*	W702R	1	K_1/1_
Membrane associated sensor kinase	*envZ*	P247S	0.24–0.95	A_1/4_, K_1/1_
3-ketoacyl-CoA thiolase	*fadI*	L246 L	1	E_2/4_
Peptidoglycan DD-transpeptidase	*ftsI*	A513S	1	K_1/1_
DNA-binding transcriptional repressor	*marR*	Ins C after A1619472 (FS118)	1	A_1/4_
ABC transporter family protein	*mdlB*	A16A	1	A_2/4_
	*mlaA*	Del F42-N43	0.93	K_1/1_
Murein tripeptide ABC transporter periplasmic binding protein	*mppA*	F244F	1	K_1/1_
Mismatch repair protein	*mutL*	W390 L	0.1	E_1/4_
Outer membrane porin F	***ompF***	Ins 7 nt after G986771 (FS71)	0.04	E_1/4_
Truncated RNase PH	*rph*	Del R676	1	E_1/4_
RNA polymerase subunit β’	***rpoC***	Del T208-K213	1	E_1/4_
DNA-binding transcriptional dual regulator	*soxR*	T145S, G146 T, A147L, R148A, L149G, L150R, Del E151-N155	1	E_1/4_
DNA-binding transcriptional dual regulator	*soxS*	S2F	0.98–1	E_3/4_
Bifunctional (p) ppGpp synthase/hydrolase	*spoT*	Ins C after T3815879 (FS223)	1	E_1/4_
DNA-binding transcriptional activator	*ybaO*	T33P	1	E_1/4_

Numbers shown indicate frequency of mutation in population. *Del* = deletion, *Ins* = insertion, underlined letters and numbers indicate nucleotides and their genomic position. Population frequency indicates the mutational frequency in the sequenced population. Strains indicates the prevalence of a mutation within different replicates. A: population with previously acquired resistance to amoxicillin, E: population with previously acquired resistance to enrofloxacin, K: population with previously acquired resistance to kanamycin. X/Y: X indicates number of replicates with particular mutation, Y indicates total number of replicates that were sequenced. Mutations associated with resistance in a wild-type background are shown in **bold**. Mutations associated with the previous resistance have been omitted from the table

**Fig. 3:**
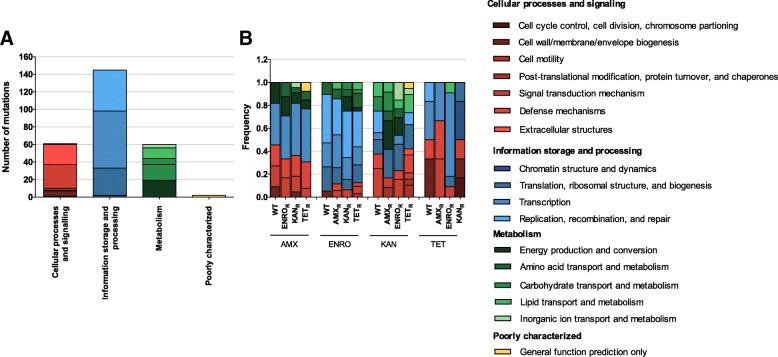
Functional annotation of mutated genes associated with antibiotic resistance. All genes containing a mutations were classified using cluster of orthologous groups (COG) classifications and grouped by the four main categories (**a**) or by resistant strain (**b**). The genes were classified into COG categories using IMG/M (https://img.jgi.doe.gov)

All genes containing a mutation were functionally annotated using clusters of orthologous groups (COG) analysis and clustered according to the four different categories (Fig. 3). The majority of mutations occur in genes involved in information storage and processing in general (145/268) and transcription (65/268) (Fig. 3a). For development of resistance to amoxicillin, enrofloxacin, and kanamycin, no major shift in the function of the mutated genes could be detected (Fig. 3b). For tetracycline, the total number of mutations is significantly lower than the numbers observed with the other three antibiotics, making the comparison slightly problematic.

**Fig. 4:**
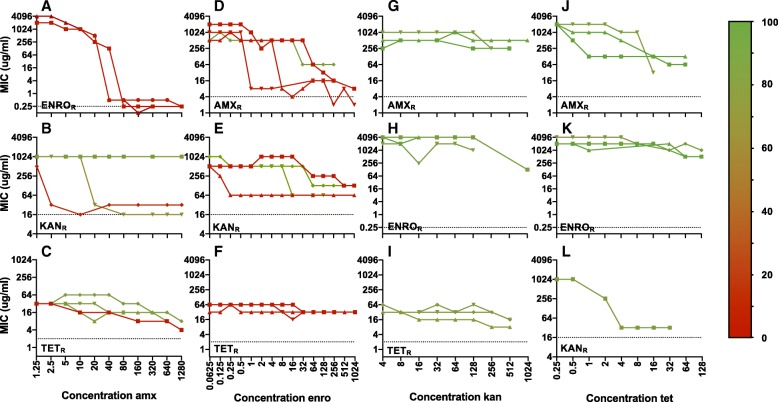
MIC values over time in resistant strains exposed to a second antibiotic. Plots show MIC (ug/ml) of the first de novo acquired resistance, indicated on the X-axes, when exposed to amoxicillin (amx, **a**-**c**), enrofloxacin (enro, **d**-**f**), kanamycin (kan, **g**-**i**), or tetracycline (tet, **j**-**l**). The dotted black lines indicate the MICs for wild-type *E. coli* MG1655. Different lines in each plot indicate the different replicates. The percentage of residual mutations was determined by dividing the number of original mutations still present at the end of the experiment by the number of mutations observed in the beginning and multiplying this number by 100. The percentages are indicated in the plots using colors based on the gradient shown on the right

The minimal inhibitory concentration (MIC) of the first antibiotic was measured in all strains exposed to two antibiotics sequentially, to determine if the original resistance could be maintained during development of resistance to a second antibiotic (Fig. 4). In general, the ability of *E. coli* to maintain its original resistance appears to depend on the combination of antibiotics used. Amoxicillin resistance is compatible with kanamycin and tetracycline exposure, but not with enrofloxacin. For enrofloxacin resistance, the same applies. Acquired resistance to kanamycin resistance can co-exist with enrofloxacin resistance, but not with resistance to other antibiotics. Tetracycline resistance persists after exposure to kanamycin, but not to enrofloxacin. Exposure to amoxicillin results in a mixed pattern.

The percentage of residual mutations was determined by dividing the number of original mutations still present in the strain at the end of the experiment by the number of mutations acquired by the parental strain and multiplying this number by 100 (Fig. 4). When comparing the residual MIC with the number of original mutations still present for most antibiotic combinations, loss of resistance can be correlated with loss of mutations and vice versa. However, a clear correlation cannot always be established. For amoxicillin and enrofloxacin resistance, the absence of mutations correlates to the loss of the original resistance. Resistance to kanamycin and tetracycline, in contrast, is partially or fully maintained, but this does not coincide with the maintenance of the original resistance mutations, suggesting that either the additionally acquired mutations also contribute to the original resistance, or that subsequent adaptation on a gene expression level maintains the level of resistance to the first antibiotic.

## Discussion

Taken together, the data presented in this study indicate that the mutations involved in de novo acquisition of resistance to each antibiotic are unique for each specific antibiotic. The shared killing mechanism proposed for all bactericidal antibiotics [6, 7] and the reduction of ROS levels measured in resistant strains [8] suggest that common mutations may occur during adaptation. However, no common mutation shared by all populations resistant to one of the bactericidal antibiotics could be identified. The one common mutation identified throughout the dataset is a *mutL* W390 L mutation (Tables 2, 3, 4 and 5). In *E. coli*, MutL is essential for mismatch repair [18]. Defects in mismatch repair are often observed in antibiotic-resistant strains [19, 20] and are known to increase mutation rates over 200-fold [21]. Considering its role in DNA mismatch repair, the mutation frequency varying between 0.09–0.20 most likely provides the population with a source of increased variation without causing deleterious effects on the entire population.

In all resistant strains, most shared mutations that are acquired by both the wild-type and strains with acquired resistance mostly have a frequency of (close to) 1, indicating fixation in the population. For each antibiotic, mutations previously associated with that antibiotic resistance were observed (Table 1). The occurrence of the same mutations in replicates concurs with the earlier observation that while the initial mutations during the development of resistance may vary, at the higher concentrations a single mutation or set of mutations becomes dominant [1]. From a mechanistic point of view, acquired antibiotic resistance can be classified into three groups: 1) prevention of access to target by reduction of permeability (*ompR* for amoxicillin, *soxR* for enrofloxacin, *sbmA* and *sapF* for kanamycin, *ompF* for tetracycline) or increased efflux (acrR and soxR for enrofloxacin, *acrB* and *acrR* for tetracycline), 2) modification of target (*gyrA*, *parC*, and *parE* for enrofloxacin), or 3) antibiotic modification (*ampC* for amoxicillin). The effect of other mutations identified in this dataset is not always clear and could vary. Some mutations appear to have a non-structural function, such as mutations found in the 5′ UTR region of *rpsJ*, *gltB*, and *ybjL* or *ybjM* in strains with acquired resistance to enrofloxacin, by potentially influencing gene expression or translation [22, 23]. Mutations in sigma factors, for example *rpoD* in amoxicillin resistance, *rpoC* in tetracycline resistance, or *rpoS* during secondary adaptation to amoxicillin or tetracycline, can redirect expression of an entire set of genes [24].

Protein moonlighting is a phenomenon in which proteins, often metabolic proteins or molecular chaperones, fulfil more than one biological function [25]. Metabolic proteins from the glycolytic or glyoxylate cycle often function as moonlighting enzymes [26]. *CitF*, encoding isocitrate lyase, is a candidate for such a role.

Interestingly, a number of synonymous mutations were also identified (Tables 2, 3, 4 and 5). Synonymous mutations are now recognized to have a range of, mostly deleterious, effects on cells, including altering mRNA structure [27, 28], rate of translation [29], protein folding [30], and fitness [31]. So far, synonymous mutations contributing to antibiotic resistance development have been described in TEM-1 β-lactamase [32], *gyrA*, and *rpoB* [33].

Not all mutations identified in the resistant strains are necessarily functionally relevant. Through genetic hitchhiking, mutations can increase in frequency because they are genetically linked to mutations that do provide a survival advantage under antibiotic pressure [34, 35].

Most resistance mutations in bacteria confer a fitness cost [36], which can be reduced by the appearance of compensatory mutations [37] or additional resistance mutations [38]. *E. coli* with acquired resistance to amoxicillin, enrofloxacin, or tetracycline is able to maintain the level of resistance when cultured under the same conditions in the absence of antibiotics [5]. It is therefore likely that some of the mutations observed in this dataset do not confer resistance themselves, but compensate for the fitness cost associated with resistance mutations. In general, mutations observed in this data set either interfere directly with the target-drug interaction, or they facilitate development of resistance by adjusting other cellular processes and thereby compensating for metabolic costs.

The observation that not all mutations associated with a particular resistance in wild-type *E. coli* appeared during secondary adaptation to the same antibiotic confirms that the effect of a single mutation on phenotype or fitness can depend on the genetic background [11, 39]. For some mutations that do not appear during secondary adaptation, the associated fitness cost could be too high in that particular genetic background. Alternatively, the mutation might not be necessary for resistance. Although no cross-resistance between resistant strains from this study has been found [8], the different genetic background itself could contribute to the ability to become resistant to a second antibiotic. Another explanation could be that the particular mutation is a compensatory mutation that is no longer necessary because of the different genetic background [38].

The mutations identified in strains exposed to a second antibiotic do not appear to be random, as the population frequency is high (Tables 2, 3, 4 and 5) and mutations appear in replicate strains (Fig. 2). The higher number of mutations could be necessary to compensate for the mutations already present in the resistant strains, or could represent alternative pathways to adaptation to the second antibiotic. In addition, a much higher degree of variability was displayed during secondary adaptation, both in the population frequencies of the mutations (Tables 2, 3, 4 and 5), as well as in the incidence of mutations among replicates (Tables 2, 3, 4 and 5). This confirms that, in addition to the different selective strength [40, 41], the genetic background can influence the mutational trajectories available [11, 42].

In an antibiotic-free environment, strains with acquired resistance to amoxicillin, enrofloxacin, and tetracycline maintain this resistance for at least two weeks after daily passaging [5]. When transferred to an environment with a different antibiotic, depending on the antibiotic combination, the original resistance is lost, partially maintained or fully preserved (Fig. 4). This suggests that the cellular adjustments necessary for adaptation to a new antibiotic are not always compatible with the earlier acquired mutations, which can have implications for the de novo development of multidrug resistance.

During resistance development, adaptation to one antibiotic can result in an increased or decreased susceptibility to other antibiotics, a concept known as collateral sensitivity or collateral resistance. This is a concept often employed to develop strategies to limit resistance development [43–47], but is not observed in the resistant strains generated in this study [8] and can therefore not explain the drug-specific effect on loss or maintenance of resistance. Similar experiments in *Pseudomonas aeruginosa* show a very similar pattern on resensitization of antibiotic resistant strains, where the effect clearly seems to depend on the combination of the antibiotics, but no explanation is available as of yet [48] .

The loss of resistance is not always correlated with the loss of the associated mutations. Resistance to kanamycin and tetracycline (Fig. 4) appears to be partially maintained, but no mutations associated with resistance could be identified in those samples. The residual resistance could be explained by the genetic background contributing to resistance, such as *gyrB* during adaptation to kanamycin and enrofloxacin, or *acrR* during adaptation to enrofloxacin or tetracycline, although no cross-resistance between resistant strains has been observed [8]. Alternatively, adaptation on a gene expression level could contribute to residual levels of resistance.

## Conclusion

The acquisition of drug resistance involves changes at various levels of cellular organization, including the genome, transcripts, and metabolites, highlighting that a complex interaction network is involved [49]. The data presented in this study support this notion. Development of resistance in strains with a previously acquired resistance does not result in the accumulation of the same mutations observed during primary adaptation in wild-type *E. coli*. This observation suggests that the genetic background of the strain itself plays an important role in resistance development. Moreover, mutations alone cannot always fully explain the resistance pattern observed, supporting the earlier suggested role for adaptation on the gene expression level in de novo acquisition of antibiotic resistance [50].

## Methods

### Bacterial strains, growth media, antibiotics, and MIC measurement

In all experimental tracks inducing resistance by exposing cells to step-wise increasing concentrations of the antibiotic [5], *E. coli* MG1655 wild-type strains were used as starting point. The choice of starting concentrations was based on MIC measurements.

Evolution experiments were performed as follows: wild-type *E. coli* was exposed to either 1.25 μg/ml amoxicillin, 0.0625 μg/ml enrofloxacin, 4 μg/ml kanamycin, or 0.5 μg/ml tetracycline. Cells were grown overnight at 37 °C, and OD_600_ were measured after 24 h. When the OD_600_ of the exposed culture was at least 75% of the OD_600_ of a reference culture that was not exposed to antibiotics, the population was considered adapted and the antibiotic concentration was doubled. Using the exposed population, new flasks were re-inoculated to a final OD_600_ of 0.1, with the flasks with the lower concentration now serving as the reference culture. The initial adaptation to each antibiotic was performed in duplicate.

After development of resistance to one antibiotic, two duplicates of each duplicate strain were used for exposure to a second antibiotic, resulting in four strains with an identical exposure history, as depicted in Fig. 1. To re-start the evolution experiments, flasks were re-inoculated with one of the resistant strains to a final OD_600_ of 0.1. Strains with acquired resistance to one or two antibiotics were compared [8]. When a population was considered adapted, glycerol stocks were made and stored at -80 °C. When relevant, cells from glycerol stocks were plated on LB agar and grown in Evans medium with the appropriate antibiotic.

Stock solutions of amoxicillin, enrofloxacin, kanamycin, or tetracycline (10 mg/mL) were filter sterilized and stored at 4 °C for maximally 2 weeks. Minimal inhibitory concentrations (MICs) were determined as described before [51]. Measurements were performed in 96-well ThermoScientific Multiskan FC spectrophotometer plate readers, shaken and at 37 °C in a final volume of 150 μl with a starting OD_595_ of 0.05. Antibiotic concentrations increasing by a factor of 2 and ranging from 0.0625 to 4096 μg/ml were applied. The lowest concentrations that limited final OD after 23 h to 0.2 or less was reported as MIC.

### Whole genome sequencing

Glycerol stocks of selected strains were plated on LB agar, and over 100 single colonies were combined to grow a liquid culture (Evans minimal medium) in the presence of the antibiotic. The culture was still growing when samples were taken to freeze at -80 °C. We then used roughly 10^9^ cells (corresponding to 2 mL of a culture with an OD600 of 1) for genomic DNA isolation. Genomic DNA was isolated using the DNeasy blood and tissue kit (Qiagen). DNA was isolated from the wild-type, the duplicates of the single exposure strains and all four replicates of the double exposure strains. A wild-type *E. coli* MG1655 was submitted to the same protocol and used as control. In this control two point mutations were observed when compared to the MG1655 reference strain. These point mutations were observed in all strains and are not reported.

gDNA libraries were generated according to the manufacturers’ protocols using the Ion Xpress™ Plus gDNA Fragment Library Preparations (Thermo Fisher Scientific). Bar-coded libraries were prepared according to the Ion Plus fragment library kit (Thermo Fisher Scientific) and the Ion Xpress DNA bar-coding kit (Thermo Fisher Scientific) according to the 200-base-read Ion Proton libraries instructions of the manufacturer. The size distribution and yield of the barcoded libraries were assessed using the 2200 TapeStation System with Agilent High Sensitivity D1000 ScreenTapes (Agilent Technologies). Sequencing templates were prepared on the Ion Chef System using the Ion PI Hi-Q Chef Kit (Thermo Fisher Scientific). Sequencing was performed on an Ion Proton System using a Ion PI v3 chip (Thermo Fisher Scientific) according to the instructions of the manufacturer.

For quality control procedures, the FASTQ files of individual samples were assessed with fastqc (http://www.bioinformatics.babraham.ac.uk/projects/fastqc/). Several quality metrics (sequencing depth, read length distribution, read quality distribution, mean read quality along the read, base frequency at each read position) were compared across samples in relation to the experimental factors using in-house software based on samtools and R (https://www.r-project.org/). To map all accepted reads to the *E. coli* K-12 MG1655 reference genome, Tmap was applied (https://github.com/iontorrent/TMAP/blob/master/doc/tmap-book.pdf). The Ion Proton system generates sequencing reads of variable lengths, and Tmap combines a short read algorithm [52] and long read algorithms [52, 53] in a multistage mapping approach. The genes were classified into COG categories using IMG/M (https://img.jgi.doe.gov).

The average sequencing depth was 226. Torrent Variant Caller (Thermo Fisher Scientific) was used to identify deviations, such as single-nucleotide polymorphisms, insertions, and deletions from the reference genome. The Torrent Variant Caller is based on Freebayes [54] and capable of somatic variant calling. Overall, parameters such as the minimum phred-scaled call quality, the minimum coverage, and the maximum strand bias, were set such that variants were called with relatively high reliability, at the cost of sensitivity. On the other hand, the minimum observed allele frequency required for a non-reference variant call was set relatively low (5%) to enable detection of low frequency events in the bacterial populations. Mutation frequency was calculated as the ratio of the number of reads containing a genetic variation to the overall read number. To differentiate between sequencing errors and true mutations, genetic deviations were excluded if either of the following conditions applied: 1) the mutations appeared in a homopolymer region, or 2) the Phred quality score was < 30 and the same mutation was not identified in a different sample with a Phred quality score of ≥30. Mutations also present in the wild-type were excluded from the analysis.

Sequencing reads were mapped directly to the MG1655 genome to enable detection of mutations and short indels. For the detection of long inserts the single nucleotide polymorphism (SNP) calling algorithm was used. Locations with a high number of called mutations were assumed to be alignment artifacts due to inserts in the genome under study and were detected with the cn.mops package [55]. The regions containing these alignment artefacts were subsequently amplified with PCR and sequenced by Sanger sequencing to achieve a more reliable analysis than the alternative, de novo assembly of the entire genome, would allow [12].

## Abbreviations

COG: clusters of orthologous groups
MIC: minimal inhibitory concentration
ROS: reactive oxygen species
SNP: single nucleotide polymorphism
